# Biotechnological Applications of Nanoencapsulated Essential Oils: A Review

**DOI:** 10.3390/polym14245495

**Published:** 2022-12-15

**Authors:** Patrícia Melchionna Albuquerque, Sidney Gomes Azevedo, Cleudiane Pereira de Andrade, Natália Corrêa de Souza D’Ambros, Maria Tereza Martins Pérez, Lizandro Manzato

**Affiliations:** 1 Research Group on Chemistry Applied to Technology (QAT), School of Technology, Amazonas State University, Manaus 69050-020, Brazil; 2 Laboratory of Synthesis and Characterization of Nanomaterials (LSCN), Federal Institute of Education, Science and Technology of Amazonas, Manaus 69075-351, Brazil

**Keywords:** pharmaceutical applications, food applications, nanocarriers, biopolymers

## Abstract

Essential oils (EOs) are complex mixtures of volatile and semi-volatile organic compounds that originate from different plant tissues, including flowers, buds, leaves and bark. According to their chemical composition, EOs have a characteristic aroma and present a wide spectrum of applications, namely in the food, agricultural, environmental, cosmetic and pharmaceutical sectors. These applications are mainly due to their biological properties. However, EOs are unstable and easily degradable if not protected from external factors such as oxidation, heat and light. Therefore, there is growing interest in the encapsulation of EOs, since polymeric nanocarriers serve as a barrier between the oil and the environment. In this context, nanoencapsulation seems to be an interesting approach as it not only prevents the exposure and degradation of EOs and their bioactive constituents by creating a physical barrier, but it also facilitates their controlled release, thus resulting in greater bioavailability and efficiency. In this review, we focused on selecting recent articles whose objective concerned the nanoencapsulation of essential oils from different plant species and highlighted their chemical constituents and their potential biotechnological applications. We also present the fundamentals of the most commonly used encapsulation methods, and the biopolymer carriers that are suitable for encapsulating EOs.

## 1. Introduction

The investigation of aromatic and medicinal plants is constantly expanding due to the current demand for natural products [1], whose benefits are directly related to the compounds produced by these plants through their secondary metabolism [2]. Essential oils (EOs) are plant secondary metabolites, and are also known as volatile oils, ethereal oils or essences [3]. They are defined as hydrophobic fluids that contain substances or compounds with “volatile aroma”, and which are extracted from plant parts, including flowers, buds, leaves and bark, and have a characteristic aroma [4]. EOs are fundamental for the survival mechanisms of plants, and play an important role in protecting them against bacteria, viruses, fungi, and herbivores, and can also attract pollinating insects and seed dispersers [5].

EOs are complex mixtures of volatile substances, including terpenes, terpenoids and phenols. This composition allows a wide spectrum of applications such as in food, agricultural/environmental, cosmetic and pharmaceutical industries [6], mainly due to their antioxidant, anxiolytic, antidepressant, anti-inflammatory, antimicrobial antibacterial, antiviral, antifungal, anti-aflatoxigenic, anticancer, antihyperglycemic and other properties [7,8,9,10]. In addition, they do not present health risks when associated with the use of synthetic pesticides [11].

However, efforts to develop new technologies, applications or functional agents based on EOs encounter challenges related to the loss of biological constituents due to high volatility, high risk of deterioration/oxidation, dependence on seasonality, limited stability and/or reduced efficacy of their biotechnological properties [12]. Nanoencapsulation, therefore, seems to be an interesting approach for minimizing these limitations, as it not only prevents the exposure and degradation of EOs and their bioactive constituents, thus creating a physical barrier, but it also facilitates their controlled release, which results in greater bioavailability and efficacy [13]. Several methods of nanoencapsulation have been developed, but those using nanoparticles or the nanoemulsions (a nanostructure system in which the encapsulation takes place) seem to be the most suitable and promising, and are therefore the most commonly explored in the current context [14].

Encapsulation processes require bioactive components to be coated within a matrix (i.e., a synthetic or natural polymer), which isolates the active ingredients from the external environment [15]. In this review, we focus on selecting studies from the last five years whose objective was to nanoencapsulate essential oils of various plant species; then, we list the main chemical constituents found in each EO and the encapsulation methods and biopolymer carriers used in the process. In addition, we highlight the biotechnological potential of nanoencapsulated EOs based on their bioactive properties and the applications developed in the selected studies.

## 2. Nanoencapsulation

Nanotechnology represents a revolutionary path for technological development in regards to the management of materials on a nanometric scale (one billion times smaller than one meter) [16]. The use of nanotechnology in natural products has gained prominence in many studies since it has resulted in effective alternative products for a number of purposes. Natural assets such as essential oils are metabolites that are of interest to various industries, but they have peculiar characteristics such as the volatility of their chemical constituents, which is why they appear as active ingredients of various encapsulated systems. Once their bioactive properties have been proven, it is necessary to use technological tools to circumvent the problem of high volatility and, at the same time, make the most of their bioactive power.

The technique of encapsulation of essential oils has been widely applied as a nanotechnological tool to protect these assets from the external environment, as well as modulate their release according to specific needs [17,18,19]. Encapsulation of bioactive compounds represents a viable and efficient alternative. In addition, this technique can increase the physical stability of substances, protect them from interactions with the environment, decrease their volatility, increase their bioactivity, reduce toxicity and even allow the release of assets over time in specific media [20,21].

The controlled-release mechanism consists of displacing the assets present in the nanoparticles (NPs) to the application medium. This displacement is gradual and is related to the concentration that is released over time, bringing benefits such as reduced evaporation of volatile assets, easy handling, reduction in phytotoxicity and environmental pollutants, which results in advantages for both the ecosystem and human health [22,23].

**Figure 1:**
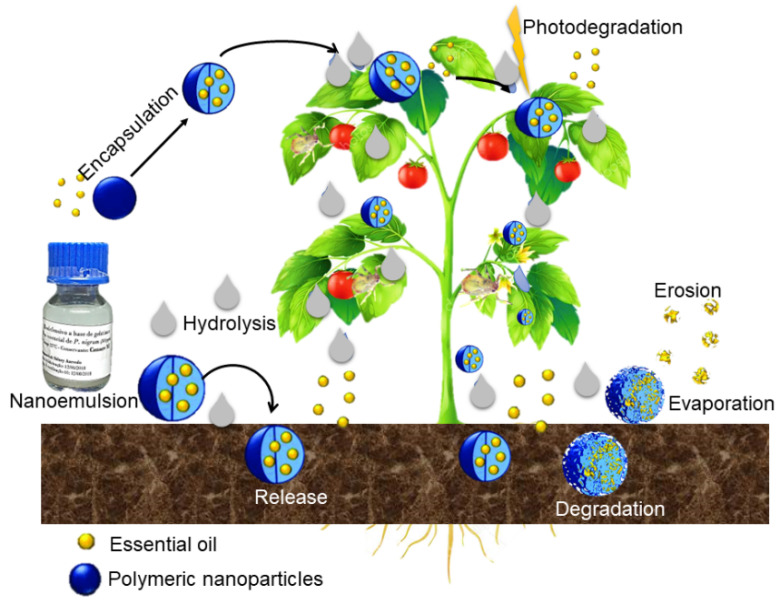
Scheme of the controlled release of essential oil and the degradation of polymeric nanoparticles. The nanosystem comprises the active ingredient encapsulated by a biodegradable polymer. Controlled release occurs due to the degradation of the polymer. When the polymer interacts with the environment in which the release takes place, there is a breakdown of the wall material, which can be due to thermal degradation or photodegradation, and then the release of the active ingredient occurs.

The investigation of the bioactive release profile in polymeric NPs provides important information about the mechanisms that guide this action. There are several possible mechanisms of bioactive release: release due to erosion or degradation of polymers; self-diffusion through pores; release through the erosion of the surface of the polymer and pulsed delivery initiated by the application of a magnetic field [24,25]. Studies of release mechanisms in nanoparticles conducted by Yasmin et al. [25] showed that the asset can be diffused through the polymer wall, and release occurs by diffusion or erosion of the matrix. Moreover, the asset can be released through the slow degradation of the polymer wall, or by the cleavage of the matrix through the action of enzymes. Controlled release can be schematized illustratively, as shown in Figure 1.

Several types of materials have been used as carriers of these active ingredients, principally natural and synthetic polymers [18,20,21,26]. Carriers, also known as wall materials, are the materials responsible for protecting the asset. During the development of encapsulating nano- or microparticles, they act as a protective membrane of the asset. In the case of colloidal systems, the carriers protect the asset from the aqueous medium. However, it is necessary to have a profound knowledge of the characteristics of the carriers in terms of biodegradability, capacity for surface functionalization, conjugation, complexation, encapsulation capacity and chemical affinity with the active substance [16,27].

Nanoencapsulated systems cover applications such as those that are biodefensive (being directly used in the control of pests and disease vectors) [20,28], in medicine (especially in the selective delivery of drugs) [29,30,31,32,33], in cosmetics (in the protection of substances prone to oxidation and in the delivery of active substances in deeper layers of the skin) [34], in food technology (protecting highly antioxidant substances and vitamins) [35,36], and in the most diverse applications in which it is necessary to guide the active ingredient to the site of action, as well as control its release rate. This type of technology allows one to maintain the characteristics and properties of active compounds, such as their protective capabilities, stabilization and prolonged release.

The encapsulation of natural assets, such as essential oils, in general, has been developed by a number of researchers in order to improve chemical stability, increase the activity of these substances and reduce volatilization, thus improving their biological potential. In this context, we can cite some examples of encapsulation of essential oils and their most recent applications.

Xavier et al. [26] encapsulated the essential oil from *Cinnamodendron dinisii* using zein as a carrier, applied in a chitosan matrix to produce an active nanocomposite film packaging for food preservation. The chitosan films obtained and functionalized with the nanoparticles demonstrated antioxidant and antimicrobial activity and were efficient in preserving ground beef. Campelo et al. [27] developed a new pharmaceutical form of semi-solid dosage based on essential oil from cloves and polysaccharides for the treatment of vaginal candidiasis. The nanoemulsions showed excellent colloidal stability and adequate pH for this specific application.

Corrado et al. [37] encapsulated essential oil from oregano in nanoparticles based on polyhydroxybutyrate and poly-3-hydroxybutyrate-co-hydroxyhexanoate using a solvent evaporation technique and achieved an encapsulation efficiency of greater than 60%. The nanoparticles obtained showed a regular distribution with a size range of 150–210 nm. When comparing the effectiveness of pure EO with encapsulated EO, it was possible to observe that encapsulated EO has greater bioactivity against microorganisms such as *Micrococcus luteus*. The authors emphasize the importance of nanoencapsulation of volatile bioactive compounds in biodegradable polymer matrices and conclude that the results pave the way for the effective exploitation of nanosystems developed for active packaging.

Azevedo et al. [28] developed an environmentally-friendly nanoparticle system for the encapsulation of the essential oil from *Piper nigrum* using gelatin and poly(ε-caprolactone) (PCL) as carriers. By evaluating the encapsulation efficiency, electrical conductivity, turbidity, pH and organoleptic properties (color and odor) after the addition of different preservatives, the authors studied the stability of the formulation generated. In this research, a particle size of between 114 ± 3 nm and 519 ± 13 nm and encapsulation efficiency of 98 ± 2% were obtained. Due to the pronounced bioactivity of the encapsulated essential oil, they concluded that the developed system has potential as a stable alternative product and as a controlling agent.

### 2.1. Nanoparticles

Nanoparticles can be formed from composites and are developed through the association of two materials that together give rise to a third material with properties superior to the separate forming components [16]. They can be called carriers and consist of multilayers that are superimposed according to the desired application under the study. Its formation depends on a number of factors, the main one being the chemical and physical interaction between the layers and the encapsulated material so that the stability of the system is achieved [31,38].

**Figure 2:**
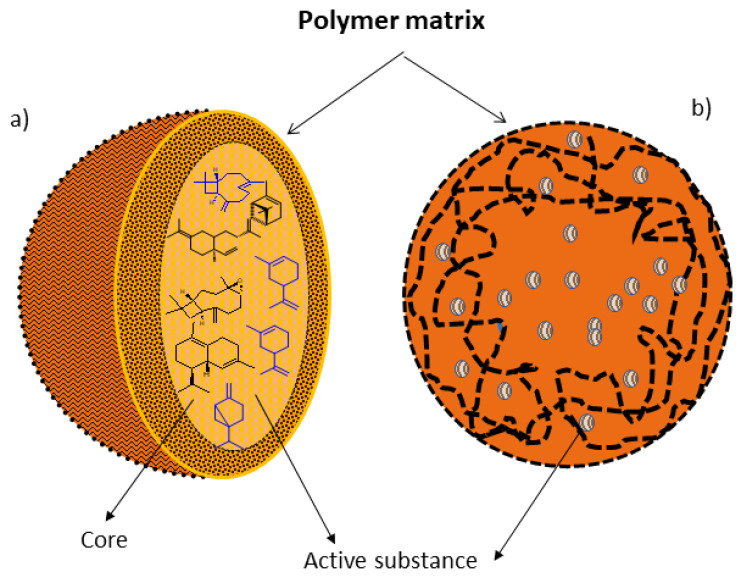
Polymer nanoparticles. (**a**) Nanocapsule: active ingredients dissolved in the matrix core and (**b**) Nanospheres: active ingredients dissolved throughout the polymer matrix. In the nanocapsules, the active substance is in the nucleus and is surrounded by a polymeric membrane. In the nanosphere, the active substance is dispersed in the polymeric matrix, and therefore it does not have a defined nucleus.

Nanoparticles, when they have a structured inner layer, are called nanocapsules and, when they have a continuous matrix, they are called nanospheres [29] (Figure 2). Nanocapsules are particles consisting of a polymeric wall containing a cavity inside, by which the active ingredient is adsorbed, though this may also be present in the polymeric wall; while nanospheres are formed by a polymeric matrix in which chemical components can be retained or adsorbed [30].

The development of polymer nanosystems is rapidly expanding and plays a key role in various areas, from pollution control to environmental technology, from electronics to photonics, from medicine to biotechnology, from materials to sensors, and so on. Several reports in the literature emphasize the growing interest in this area. This trend is based on the unique properties of polymer nanosystems, which meet numerous applications and market needs [31,32].

The combination of biopolymer materials represents an important alternative for encapsulating essential oils. However, understanding the nanoparticle development project is crucial for obtaining stable formulations, adequate controlled-release and surface functionalization for further conjugation with bioactive molecules or ligands. The development of these encapsulating nanoparticles involves a series of parameters that are related to the specific purpose of action of the encapsulated substances and also to the controlled release mechanisms, which can often be related to the type of carrier used [33,34,35,36]. Its formation depends on certain factors, the main one being the chemical and/or physical interaction between the layers and the encapsulated active substance.

Encapsulated systems are efficient strategies for transporting the active substance to its site of action through the choice of a carrier and the appropriate route, with the main objectives being protecting its content from environmental factors (light, moisture, oxygen and interactions with other compounds), in addition to controlled release and release under stimuli (such as changes in pH, physical disruption, swelling, dissolution, etc.). In addition, encapsulation can also mask the unpleasant taste and/or odor and increase the acting time of the active compound, thus prolonging its effect. In this case, the type of nanoparticle and the place where the active substance will be exposed (adsorbed on the surface or not) will depend on the desired final characteristics, such as application, size, size distribution, degree of biodegradability and compatibility of the polymer with the active substance [18].

### 2.2. Development of Nanoparticles

Several methodologies exist for the development of encapsulation of polymeric nanoparticles, which in their formulations generally employ materials such as polymers (synthetic or natural), surfactants, bioactive compounds, organic solvents and essential oils, depending on the formulation. For the preparation of nanoparticles with the purpose of carrying natural active ingredients, the physicochemical properties of the polymer must be taken into account. Polymers and their degradation products must be biocompatible and biodegradable, and cause no harm or impact to the environment [38,39].

According to Mora-Huerta et al. [33], the following classic methods of obtaining nanoparticles are generally used: nanoprecipitation, emulsion-diffusion, double emulsification, emulsion coacervation, polymer coating and layer-by-layer.

**Figure 3:**
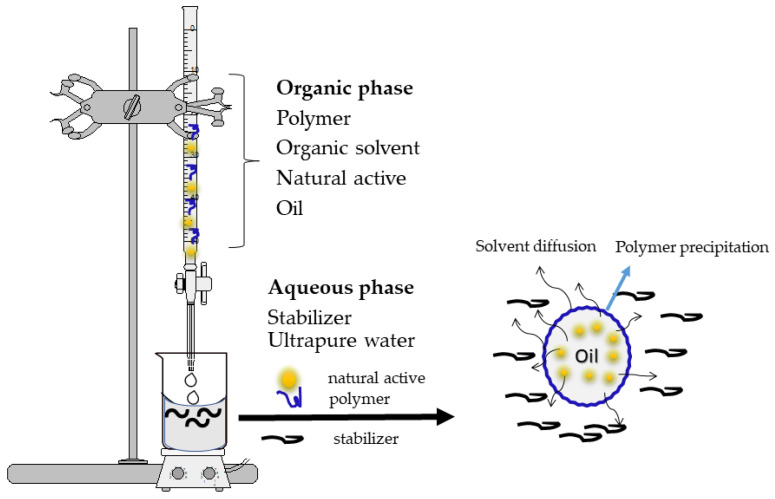
Formation of polymeric nanoparticles using the nanoprecipitation method. The organic phase consists of a solution—or a mixture—of a polymer (carrier), the organic solvent, the active substance (natural active ingredient), oil and a lipophilic surfactant. The aqueous phase consists of a mixture of surfactant (stabilizer) and ultrapure water. Using a burette and a magnetic stirrer, the organic phase is slowly added to the aqueous phase.

The nanoprecipitation method (Figure 3) is also called solvent displacement or interfacial deposition. This method requires two phases, one organic and one aqueous. The organic phase essentially consists of a solution or a mixture of solvents (ethanol, acetone, hexane, dichloromethane, etc.), of a macromolecule with a carrier role (synthetic, semisynthetic or natural polymer), the active substance, oil and a lipophilic surfactant. However, the aqueous phase consists of a mixture of surfactant in an aqueous medium. In this method, the nanoparticles are obtained as a colloidal suspension that is formed when the organic phase is slowly added to the aqueous phase with moderate agitation. The main variables of the procedure are those associated with the conditions of addition of the organic phase to the aqueous phase, such as the organic phase injection rate and the aqueous phase agitation rate [33,34,40,41].

**Figure 4:**
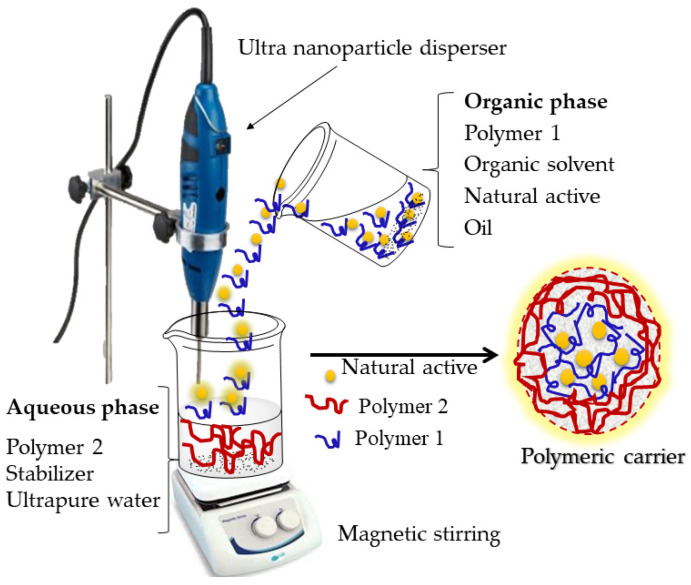
Formation of carrier systems using the emulsion–diffusion method. The organic phase contains the polymer, the organic solvent, the natural active ingredient, and the oil. The aqueous phase contains a different polymer, a stabilizer and the ultrapure water. The particles are formed by polymer precipitation along with the encapsulation of the natural active ingredient.

The preparation of nanoparticles using the emulsion–diffusion method (Figure 4) allows the nanoencapsulation of lipophilic and hydrophilic active substances.

The procedure requires three phases: organic, aqueous and dilution. When the goal is the nanoencapsulation of a lipophilic active substance, the organic phase contains the polymer, the active substance, the oil and an organic solvent that is partially miscible with water. The organic phase is emulsified under vigorous stirring in the aqueous phase and, after primary emulsion formation, the organic solvent is diffused to the external aqueous phase by adding excess water (dilution), which leads to polymer precipitation and nanoparticle formation [33,39].

The nanocapsule formation mechanism suggested by Quintanar-Guerrero et al. [42] is based on the theory that each emulsion droplet produces several nanocapsules, and that these are formed by the combination of polymer precipitation and interfacial phenomena during solvent diffusion [43].

The double emulsification method consists of the formation of two emulsions that are usually prepared using two surfactants: a hydrophobic one intended to stabilize the water/oil interface of the internal emulsion, and a hydrophilic one to stabilize the external interface of the oil droplets for water/oil/water emulsions. The primary emulsion is formed with the use of ultrasound, and the hydrophobic surfactant stabilizes the water/oil interface of the internal phase. The secondary emulsion can also be formed with ultrasound, and the dispersion of the nanoparticles is stabilized by the addition of another surfactant (hydrophilic) [33,39,40].

The emulsion coacervation method involves the formation of an oil/water emulsion, in which the organic phase is composed of the solvent and the bioactive compound, and the aqueous phase is composed of the polymer, a stabilizing agent and water. The emulsion can be formed by the use of ultrasound or mechanical stirring. Then, the coacervation process is carried out by adding electrolytes, adding a water-immiscible solvent or dehydrating agent, or changing the temperature. Finally, the coacervation process is supplemented with additional measures for the formation of lattices, which makes it possible to obtain the nanoparticles. The formation of nanoparticles occurs during the coacervation phase, in which there is precipitation of the polymer from the continuous emulsion phase to form a film that agglomerates into nanoparticles [31,33,39,40].

The polymer-coating method is used for the deposition of a thin polymer layer on the surface of the nanoparticle that was previously formed by the adsorption of the polymer on uncoated nanoparticles when incubated with a polymer solution under stirring. Likewise, this polymeric layer can be added during the final phase of the methods mentioned above [33,39].

The layer-by-layer method favors the acquisition of vesicular particles, which are also called polyelectrolyte capsules. The mechanism of formation is based on irreversible electrostatic attraction that leads to the adsorption of polyelectrolytes in the formed layers. A polymer layer is adsorbed by incubation in the polymer solution, which decreases the solubility of the polymer by dropwise addition of solvent. This procedure is then repeated with a second polymer and several polymer layers are deposited sequentially [33].

## 3. Chemical Composition of Essential Oils

Generally speaking, constituents of EOs mainly comprise terpenes, phenylpropanoids, straight-chain compounds and diverse groups. Among these, terpenes are the most abundant compounds and comprise hydrocarbons of the class of mono, sesqui and diterpenes; and oxygenated compounds, such as alcohols, oxides, aldehydes, ketones, phenols, acids, esters and lactones [44,45].

The chemical composition of EOs varies between the different plant species that produce them. Among the studies selected for this review, 20 different botanical families were explored, with emphasis on Lamiaceae, Myrtaceae, Lauraceae, Apiaceae and Rutaceae, in which the species *Thymus vulgaris* and *Eugenia caryophyllata* are the most cited. Other plants, such as *Origanum* sp. and *Cinnamomum* sp., are also extensively used for EO extraction, and were found within the articles selected for this review. Some of the studies address EOs that present main components that correspond to more than 50% of their chemical composition, such as the EO of *Aniba canelilla* (1-nitro-2-phenylethane = 86.63%) [46], *A. rosaeodora* (linalool = 81.46%) [47], *Cymbopogon citratus* (citral = 67.4%) [48], *Pimpinella anisum* (anethole = 51.02%) [49], *Mentha pulegium* (pulegone = 72,18%) [50], *Syzygium aromaticum* (eugenol = 71.92%) [51], *C. nardus* (citral = 62.73%) [52], *Illicium verum* (anethole = 89.12%) [53], *T. capitatus* (carvacrol = 76.1%) [54], *Kaempferia galanga* (ethyl-*p*-metoxycinnamate = 59.4%) [55], *Cinnamomum tamala* (linalool = 82.64%) [56], *Foeniculum vulgare* (anethole = 73.27%) [57], and *Coriandrum sativum* (linalool = 65.18%) [58].

In addition, it is interesting to mention the case of the EO of *E. caryophyllata,* which is reported in the works of De Hasheminejad et al. [59]; Hadid et al. [60]; and Kujur et al. [61], and for which the main component is eugenol, with 77.2%, 89.86% and 73.6% of eugenol being found in the EO, respectively. In other cases, in the same botanical species, there may be different main components when the EO is characterized by different authors, and data indicate that the chemical composition may vary according to the season, growing conditions and the part of the plant used in the process of obtaining the EO [62].

The methods of extracting essential oils vary according to the state in which the plant is found [63,64]; thus, for each purpose of the oil, a different technique can be chosen. Among the techniques for the extraction of essential oils, hydrodistillation, pressing, solvent extraction, enfloration, supercritical gases and microwaves can be used [65,66]. In hydrodistillation, the constituents of the plant material are dragged by water vapor because they have a higher vapor pressure than water. This method is most often used for extracting EOs from fresh plants. Pressing, on the other hand, is a technique used to extract EOs from citrus fruits. The pericarps are pressed and the layer containing the EO is separated. Extraction with non-polar solvents, in turn, generates a product of low commercial value, since other lipophilic compounds are extracted along with the EO. In enfloration, on the other hand, the product has high commercial value, as it is used to obtain the EO from petals. It is carried out with the help of a fat, at room temperature, for a short period. Extraction with supercritical gases allows one to recover natural aromas of various types, and not only the essential oil. It is a very efficient method and is ideal for industrial extraction of EOs. Microwave-assisted extraction combines microwaving with traditional solvent extraction. Selective heating during extraction increases process kinetics and yield [64].

The chemical compounds found in EOs give them their biotechnological properties, which can be applied in different commercial areas. In the food industry, EOs are employed as alternative functional ingredients to extend the shelf life of food products, thus ensuring microbial safety by preventing the development of pathogens such as *Salmonella* spp. and *Lysteria* spp. [67,68]. They also act as antioxidants and preservatives in food, and can be incorporated into packaging [69,70], in addition to representing a potential natural alternative to the use of chemical preservatives [71].

The chemical composition of EOs also confers the application of EOs in the pharmaceutical industry, such as the EO of *Melissa officinalis*, whose in vitro cytotoxicity assay indicated that this oil can be effective against a number of human cancer cell lines (A549, MCF-7, Caco-2, HL-60, K562) [72].

Since EOs are rich in volatile bioactive substances, the nanocapsulation technique has been reported as an important ally in the biotechnological application of these metabolites, thus increasing their efficiency and preventing their degradation in the short term.

## 4. Biotechnological Potential of Nanoencapsulated Essential Oils

Encapsulation of EOs can be developed at micro or nano levels and presents several possibilities for biotechnological applications. However, nanoencapsulation technology has been growing exponentially and is now being used in a variety of industrial applications, such as textiles, the food industry, cell immobilization, fermentation processes, drug delivery, cell transplantation, agriculture, and cosmetics, among others [73]. In this review, we will focus on nanoencapsulated EOs with promising applications in the food, cosmetics, pharmaceutical and environmental industries. Table 1 summarizes different nanoencapsulated essential oils and highlights their chemical composition, nanoencapsulation method, the polymeric material used and their biotechnological applications.

### 4.1. Pharmaceutical Applications

The use of EOs in traditional systems of medicine has been practiced since ancient times in human history, as they exhibit different biological properties; but only recent advances and technologies have allowed the stabilization, prolonged release, targeted delivery and maintenance of these bioactive components. These advantages are conferred to nanoencapsulated EOs. In this context, the development of formulations that maintain the biological and physicochemical properties of EOs is an important choice when used as an active ingredient in pharmaceutical formulations.

Nanoencapsulated EOs can be used as a healing accelerator for infected wounds and dressing of diabetic ulcers. These actions are described in the work of Kreutz et al. [46] who developed and characterized a nanoemulsion using the EOs of leaves and branches of *A. canelilla*—an aromatic plant from the Amazon. The authors observed that the nanosystem developed is promising for the treatment of topical inflammation. This is related to its predominant chemical compound (1-nitro-2-phenylethane = 86.63%), which has already been reported as an anti-inflammatory and antinociceptive substance. The authors emphasized that nanoemulsions are the most-reported nanostructure system to encapsulate essential oils, since they allow one to incorporate high doses of these active products.

Ghodrati, Farahpour & Hamishehkar [74] also developed nanoemulsions from *Mentha* spp. and obtained bioproducts in the form of nanogels with promising antibacterial activity against gram-negative and gram-positive bacteria. The nanoemulsions showed adequate encapsulation efficiency and size distribution. It is interesting to note that the formulations accelerated the healing process of an infected wound model and this may be an appropriate strategy for producing topical healing formulations.

The healing of infected wounds was also reported in a study with the nanoencapsulated EOs of pennyroyal (*M. pulegium*) and thyme (*T. vulgaris*), which showed antimicrobial and antifungal potential, respectively. Nanoencapsulated pennyroyal EO decreases the duration of the inflammatory phase and thyme EO was strongly recommended for the treatment of cutaneous mycoses [50,75]. It is also interesting to mention the work of Rozman et al. [76] with *Homalomena pineodora* EO nanoparticles, synthesized by ion gelification, and whose pharmaceutical properties revealed a broad spectrum of activity against clinical microbial strains that infect diabetic skin lesions *(Escherichia coli*, *Proteus mirabilis*, *Yersinia* sp., *Klebsiella pneumoniae*, *Shigella boydii*, *Salmonella typhimurium*, *Acinetobacter anitratus*, *Pseudomonas aeruginosa*, *Candida albicans* and *C. utilis*). The bioactive behavior of this nanocapsule may be due to the synergistic effect of the EO with the polymeric carrier (chitosan).

Nanoencapsulated EOs have important future prospects for the treatment of various types of cancer. Recent studies have developed nanocapsules using different methods (ionic *gelidification*–emulsion, nanoprecipitation and high-speed homogenization) with EOs from Cynometra cauliflora [77], Morinda citrifolia [78], Citrus spp. [79]. and Origanum glandulosum [80], respectively, all encapsulated with chitosan, with the exception of the latter for which sodium alginate was used. These nanoencapsulated EOs showed anticancer action against human lung tumor cells A549, breast cancer MDA-MB-468, melanoma A-375, human hepatocellular carcinoma (HepG2) and human breast cancer cells MCF-7 and MDA-MB-231.

The use of nanoencapsulated EOs with antimicrobial activity has also been widely reported. In the pharmaceutical context, microbial resistance is a serious public health problem, and these EOs present remarkable potential against different pathogens. The following nanoencapsulated EOs exhibit antimicrobial activity: EO of *O. vulgare* and *T. capitatus* nanocarried with chitosan [81], EO of *Cinnamomum* spp. nanocarried with sodium alginate [82], EO of *C. aurantifolia*, *C. hystrix* and *Citrofortunella microcarpa* [83], EO of *Poiretia latifolia* [84], EO of *O. vulgare* and *T. capitatus* nanocarried with poly(ε-caprolactone) [71], EO of *C. zeylanicum*, *T. vulgaris* and *Schinus molle*, nanocarried with chitosan [85], EO of *E. caryophyllata* [86] and EO of *C. commutatus*, also nanocarried with chitosan [87].

### 4.2. Cosmetic Applications

Among the problems faced by the cosmetic industry, microbial contamination stands out as one of the most important, since it negatively affects formulations and is difficult to control [15]. To circumvent such situations, cosmetic preservatives are used, which are chemical substances of the most varied classes and which prevent the proliferation of microorganisms in the formulas, thereby increasing the shelf life of these products. However, some of these preservatives may have undesirable effects, such as causing allergies, irritations, and may even have toxic effects [88]. In an attempt to reduce these problems and increase the natural commercial appeal of cosmetics, it is possible to employ nanoencapsulated EOs in cosmetic formulations.

Recent studies have involved the basic research of nanoencapsulated EOs with antimicrobial properties, as well as the exploration of the antioxidant potential of these products for cosmeceutical purposes. This is the case of the study conducted by Hadidi et al. [60], who encapsulated *E. caryophyllata* EO loaded with chitosan by ionic gelidification. The nanoparticles had a high antibacterial activity (47.8–48 mm inhibition halo) against *L. monocytogenes* and *S. aureus*. Huang et al. [89] and Sampaio et al. [90] evaluated the encapsulation efficiency of the EO of *Cedrus deodara* (loaded with modified starch), *T. vulgaris* and *M. officinalis* (without wall material) in the form of nanoemulsions. All the nanoemulsions evaluated were stable. In addition, the antioxidant and antibacterial activity of the EOs were accentuated after nanoencapsulation.

The nanoencapsulation method using nanoprecipitation seems useful for formulations that aim to maintain the antioxidant and antimicrobial activity of encapsulated EOs, with a view to use in the cosmetics industry. This method is addressed in the studies of Sheta et al. [91] involving the EO of peppermint (*M. piperita*) and green tea (*Camellia sinensis*) encapsulated with chitosan. This methodology improved the thermal stability of the EOs with a controlled release profile for 72 h; in addition, nanoencapsulation also increased the antioxidant activity for both essential oils.

The nanoprecipitation process was also useful for nanoencapsulating *Cannabis sativa* EO using alfalfa protein as a wall material and proved to be an efficient strategy for improving the stability and functionality of the EO. After nanoencapsulation, the increase in the antioxidant activity of the EO was observed, and a product recommended for applications in the cosmetic and food areas was obtained [92].

### 4.3. Food Applications

Nanoencapsulated EOs can be used for various food applications, mainly in foods that are sensitive to oxidation or degradation under certain conditions, and which decreases the final quality of the product. In this sense, some authors were able to verify the permanence of the antioxidant activity when the nanoencapsulated EOs were used, although the methods of obtaining the encapsulated product or its polymeric matrix were distinct. Karimirad et al. [93] tested nanocapsules of *Citrus aurantium* EO obtained by nanoprecipitation; Chaudhari et al. [94] obtained nanocapsules from *Melaleuca cajuputi* EO obtained by ionic gelidification; while Arabpoor et al. [95] worked with nanocapsules loaded with EO from *Eryngium campestre*, also obtained by ionic gelidification. The polymeric matrix used by these authors was the same, i.e., chitosan, which is a biodegradable biopolymer, but they obtained the encapsulated EOs using different methods.

For applications in the food industry, the presence of antioxidant, antifungal and anti-aflatoxigenic activity in the EO is highly desired, since it can directly influence food preservation [53,96,97]. Deepika et al. [98] used the EO of *Petroselinum crispum* leaves, which were nanoencapsulated using ionic gelidification with the biopolymer chitosan, and obtained promising nanoemulsions with antioxidant, antifungal and anti-aflatoxigenic activity. The authors recommended the application of this bioproduct on an industrial scale in the management of the loss that occurs during the storage of chia seeds, which is caused by aflatoxin-producing fungi [97]. Similarly, Cai et al. [99] explored the efficacy of chitosan nanocapsules loaded with EO from *Ocimum basilicum* using the ionic gelidification–emulsion method, and their results demonstrated the strong antibacterial and antibiofilm capacity of the nanoencapsulated EOs against the pathogenic bacteria *E. coli* and *S. aureus*.

Nanoencapsulated EOs are also highly recommended for the direct coating of fruits in order to prolong their sensory characteristics. Using ionic gelidification–emulsion, Singh et al. [56] nanoencapsulated the EO of *Cinnamomum tamala* in a chitosan nanoemulsion and obtained prolongation of the shelf life of stored millet by inhibiting fungi and aflatoxins. Similarly, Antonioli et al. [48] were able to perform the in vivo control of *Colletotrichum acutatum* in apples by using nanocapsules of the EO from *C. citratus* carried in poly(lactic acid) (PLA) via nanoprecipitation. The post-harvest apples showed less bitter rot lesions after the use of the nanoencapsulated EO.

Encapsulating EOs can also help control the production of mycotoxins. Wan et al. [100] studied the ability of nanoemulsions of EO of thyme (*T. vulgaris*), lemongrass (*C. citratus*), cinnamon (*Cinnamomum* spp.), peppermint (*M. piperita*) and cloves (*E. caryophyllata*) in inhibiting mycotoxins. The authors stated that the chemical composition of the EO directly impacts their inhibitory activity. Other studies report the anti-aflatoxigenic property of nanoencapsulated EOs, either by the formulation of nanoemulsions from EO of dried fruits of *C. tamala* carried by chitosan [56], or by the formulation of nanogels based on chitosan–cinnamic acid and EO of *F. vulgare* using the nanoprecipitation method [57].

Protecting against fungi and bacteria directly prolongs the shelf life of food. Authors, such as Sagar et al. [101], have tested nanoemulsions of EOs (cinnamon, cloves and thyme) as coating materials for breaded steamed chicken. The nanoemulsions maintained the quality and sensory attributes, and it was possible to double the storage time of the product from 10 to 20 days. Hossain et al. [102] obtained chitosan-based antifungal films reinforced with cellulose nanocrystals loaded with the EO of *O. compactum*, *T. vulgaris*, *M. alternifolia* and *M. piperite* that, when combined with radiation, were efficient against the growth of fungi (*Aspergillus niger*, *A. flavus*, *A. parasiticus* and *Penicillium chrysogenum)* during rice storage, without organoleptic changes.

### 4.4. Environmental Applications

In the environmental context, it is known that science has been facing important challenges. The use of synthetic agricultural pesticides, for example, used against fungi, insects and other pests, contributes not only to environmental contamination (soil and water courses) but also contamination of the food that is produced. Nanoencapsulated EOs have been described as promising alternatives to the use of these xenobiotics and the chitosan-loaded nanocapsules formulated by the ionic gelidification method seem the most appropriate for this application. Ibrahim et al. [103] evaluated the use of EOs from *C. nardus* carried by chitosan and cellulose in the control of cotton leafworm (*Spodoptera littoralis*). The nanosystems have high toxicity and cause the interruption of the development of larvae, thus not only revealing greater insecticidal activity in the mortality of larvae and pupae, but also demonstrating the great bio-insecticidal potential of encapsulated EOs.

Rajkumar et al. [104], using the same encapsulation methods as Ibrahim et al. [103], observed the insecticidal action of the EO of *M. piperita* against pests in grain (*Tribolium castaneum* and *Sitophilus oryzae*). The inhibition caused by the polymer nanoparticles containing the EO was more effective against *Tribolium castaneum*, which indicates promising potential for the establishment of a pest management program.

Sundararajan et al. [105] used the nanoencapsulation process for the formulation of nanoemulsions with the EO of *Ocimum basilicum* (basil) leaves, whose result was efficient in terms of antimicrobial, antioxidant and larvicidal activity against third-stage *Culex quinquefasciatus* larvae. The bioproduct proved to be thermodynamically stable for controlled release and effective in combating mosquitoes. The larvicidal activity of nanoencapsulated EOs was also reported by Ferreira et al. [106], who used nanoemulsions of the EO of *Siparuna guianensis* loaded with chitosan, and by Santos et al. [53], who used nanocomposites containing the EO of *S. aromaticum*, bentonite clay and polyvinylpyrrolidone (PVP), which showed effective larvicidal activity against *Aedes aegypti* when compared to the same EO without encapsulation.

The acaricidal activity of the EO of *Satureja hortensis,* nanoencapsulated in chitosan nanoparticles, was evaluated against *Tetranychus urticae*. The authors obtained high encapsulation efficiency (greater than 95%), with effective durability and controlled release, and maintained the acaricidal activity for a long period of time (up to 25 days), thus confirming the possibility of using the nanoencapsulated system as a suitable vehicle for other acaricidal applications [107].

The materials used in nanotechnology formulations can be selected according to important characteristics for environmental applications: biodegradability, capacity for surface functionalization, conjugation and complexation [17]. Biodegradable polymers, whether synthetic or natural, are prone to degradation through natural processes [108,109]. Biopolymers, however, have the advantage of being easily degraded in the environment. Collagen, gelatin, chitosan, gums, and starches, among other biopolymers, have peculiar characteristics, such as low toxicity, cell compatibility and are biodegradable. They therefore emerge as interesting alternatives for use as a wall material in the nanoencapsulation of essential oils [110,111].

Biodegradation can occur in the presence or absence of oxygen, through the action of microorganisms that, in turn, release enzymes capable of breaking down polymer molecules into smaller chains. Finally, these fragments can get into microbial cells where they will be decomposed into CO_2_, CH_4_, H_2_O, mineral salts and biomass [109].

**Table 1:** Nanoencapsulated essential oils and their biotechnological potential.

Essential Oil				Nanoencapsulation					Biotechnological Potential	
Species	Common Name	Plant Part ^a^	Main Chemical Compounds	Encapsulation Method	Polymer Carrier	Nanoproduct Obtained	Biological ACTIVITY	Application	Industry	Nº
*Piper nigrum*	Black pepper	-	β-caryophyllene (28%); limonene (15%); sabinene (11.4%); β-pinene (11%)	Complex Coacervation	Gelatin and sodium alginate	Nanocapsules	-	-	Food	[13]
*Aniba rosaeodora*	Rosewood	Leaves	linalool (81.46%); α-terpineol (7.4%); linalool oxide (1.56%)	Ionic gelidificaton- emulsion	Chitosan	Nanoemulsions	Antifungal, anti- aflatoxigenic	Fruit coating	Food	[47]
*Cymbopogon* *citratus*	Lemongrass	Leaves	citral (67.4%); neral (25.6%); geranial (41.8%); β-myrcene (18.1%)	Nano- Precipitation	poly(lactic acid)-PLA	Nanocapsules	Antifungal	Fruit coating	Food	[48]
*Pimpinella anisum*	Aniseed	Fruits	anethole (51.02%); estragole (24.75%); fenchone (13.22%)	Ionic gelidification- emulsion	Chitosan	Nanoemulsions	Antioxidant, antifungal, anti- aflatoxigenic	Food Preservative	Food	[49]
*Cymbopogon nardus*	Citronella grass	-	citral (62.73%); geranyl acetate (9.53%); geraniol (4.52%)	Ionic gelidification- emulsion	Chitosan	Nanoemulsions	Antioxidant, antifungal, anti- aflatoxigenic	Food Preservative	Food	[52]
*Illicium verum*	Star anise	Fruit	anethole (89.12%); estragole (4.85%)	Nano- Precipitation	Chitosan	Nanocapsules	Antioxidant, antifungal, anti- aflatoxigenic	Food Preservative	Food	[53]
*Thymus capitatus*	Conehead thyme	Aerial parts	carvacrol (76.1%); y-terpinene (6.7%); β-caryophyllene (2.7%)	Nanoemulsion	-	Nanoemulsions	Antibacterial	Food preservative	Food	[54]
*Kaempferia* *galanga*	Sand ginger	Rhizomes	ethyl-*p*-methoxycinnamate (59.4%); trans-methyl cinnamate (17.1%); pentadecane (6.9%)	Nanoemulsion	-	Nanoemulsions	Antifungal	Food preservative	Food	[55]
*Cinnamomum tamala*	Indian bay leaf	Fruits	linalool (82.64%); caryophyllene oxide (3.1%); terpinen-4-ol (2.88%)	Ionic Gelidification	Chitosan	Nanoemulsions	Antifungal, anti- Aflatoxigenic	Food preservative	Food	[56]
*Foeniculum vulgare*	Common fennel	Fruits	anethole (73.27%). fenchone (6.84%); D-limonene (4.39%)	Nano- Precipitation	Chitosan- cinnamic acid	Nanogéis	Antifungal, anti- aflatoxigenic	Food preservative	Food	[57]
*Coriandrum* *sativum*	Coriander	Dried seeds	linalool (65.18%); geranyl acetate (12.06%); α-pinene (4.76%)	Ionic gelidification- emulsion	Chitosan	Nanoemulsions	Antioxidant, antifungal, anti-aflatoxigenic	Food preservative	Food	[58]
*Eugenia* *caryophyllata*	Cloves	Ground aerial part	eugenol (77.2%); eugenyl acetate (8.31%); β-caryophyllene (7.19%)	Ionic gelidification- emulsion	Chitosan	Nanocapsules	Antifungal	Food preservative	Food	[59]
*Eugenia* *caryophyllata*	Cloves	Flower buds	eugenol (73.6%); caryophyllene (9.67%); oleic acid (2.03%)	Nano- precipitation	Chitosan	Nanogels	Antioxidant, antifungal, anti- aflatoxigenic	-	Food	[61]
*Citrus aurantium*	Seville orange	Bark		Nano- precipitation	Chitosan	Nanocapsules	Antioxidant	Food preservative	Food	[93]
*Melaleuca cajuputi*	Cajuput	Leaves	α-pinene (49.24%); bornyl acetate (21.07%); camphor (11.70%)	Ionic gelidification	Chitosan	Nanocapsules	Antioxidant	Food preservative	Food	[94]
*Eryngium* *campestre*	Watling Street thistle	Leaves and roots	β-sesquiphellandrene (16.44%); isophytol (12.27%); stigmasterol (10.11%)	Ionic gelidification	Nanochitosan	Nanocapsules	Antioxidant	Food preservative	Food	[95]
*Myristica fragrans*	Mace	Dried seeds	myristicin (39.43%); methyleugenol (8.15%); safrole (6.26%)	Nano- precipitation	Chitosan- cinnamic acid	Nanogels	Antioxidant, antifungal, anti- aflatoxigenic	Food preservative	Food	[96]
*Petroselinum* *crispum*	Parsley	Leaves	carvacrol (48.45%); D-limonene (20.80%); cuminaldehyde (15.78%)	Ionic gelidification	Chitosan	Nanoemulsions	Antioxidant, antifungal, anti- aflatoxigenic	Food preservative	Food	[98]
*Ocimum basilicum*	Basil	-	eugenol (48.32%); caryophyllene (26.26%); methyl ester (5.78%)	Ionic gelidification- emulsion	Chitosan	Nanocapsules	Antibacterial, Antibiofilm	Food preservative	Food	[99]
*Thymus vulgaris Cymbopogon* *citratus* *Cinnamomum* spp. *Mentha* × *piperita Eugenia* *caryophyllata*	Thyme, lemongrass, Cinnamon, Peppermint, Cloves	-	Thyme: thymol (21.69%); *p*-cymene (21.31%); γ-terpinene (13.87%). Lemongrass: β-citral (31.33%); α-citral (14.65%). Cinnamon: eugenol (37.13%); caryophyllene (9.87%). Peppermint: menthol (29.4%); l-menthone (17.97%). Cloves: eugenol (34.42%%); eugenol acetate (24.53%%); caryophyllene (21.30%%).	Nanoemulsion	-	Nanoemulsions	Antifungal, mycotoxin inhibitor	Food preservative	Food	[100]
*Cinnamomum zeylanicum* *Thymus vulgaris Syzygium* *aromaticum*	Cinnamon, Thyme, Cloves	-	-	Oil in water emulsion	Chitosan	Nanoemulsions	Antioxidant, antimicrobial	Food preservative	Food	[101]
*Origanum* *compactum* *Thymus vulgaris Melaleuca* *alternifolia* *Mentha* × *piperita*	Compact oregano, Thyme, Tea tree, Peppermint	-	Oregano: carvacrol (46.37%); thymol (13.70%); *p*-cymene (13.33%). Thyme: thymol (26.04%); *p*-cymene (26.36%); y-terpinene (16.69%). Tea tree: terpinen-4-ol (38.4%); γ-terpinene (22.6%). Peppermint: menthol (33.38%); menthone (34.31%)	Nanoemulsion	Chitosan	Nanocapsules	Antifungal	Food storage	Food	[102]
*Zingiber officinale*	Ginger	-	-	Nanoemulsion	Carnauba wax, hydroxypropylmethylcellulose	Nanoemulsions	-	Food preservative	Food	[112]
*Salvia rosmarinus*	Rosemary	-	-	Nanoemulsion	-	Nanoemulsions	-	-	Food	[113]
*Origanum* *majorana*	Sweet marjorum	-	terpinen-4-ol (28.92%); α-terpineol (16.75%); linalool (11.07%)	Ionic gelidification- emulsion	Chitosan	Nanocapsules	Antioxidant, antifungal, anti- aflatoxigenic	-	Food	[114]
*Myristica fragrans*	Nutmeg	Seeds	elemicin (27.08%); myristicin (21.29%); thujanol (18.55%)	Ionic gelidification	Chitosan	Nanoemulsions	Antifungal, anti- Aflatoxigenic	Food preservative	Food	[115]
*Pelargonium* *graveolens*	Rose- scented geranium	Aerial parts	citronelil (19.1%); menthone (16.7%); linalool (15.1%); isomenthone (12.2%)	Oil in water emulsion	Chitosan	Nanogels	Antifungal, anti Aflatoxigenic	-	Food	[116]
*Toddalia asiatica*	Orange climber	Leaves	caryophyllene oxide (24.4%); 1.3-hexadiene, 3-ethyl-2,5-dimethyl (24.08%); 1,4,7-cycloundecatriene,1,5,9,9- tetramethyl-Z,Z,Z (9.46%)	Ionic gelidification	Chitosan	Nanocapsules	Antifungal, anti- Aflatoxigenic	-	Food	[117]
*Bunium persicum*		Seeds	cuminaldehyde (21.23%); sabinene (14.66%); γ-terpinene (12.49%)	Nanoemulsion	Chitosan- cinnamic acid	Nanogels	Antifungal, anti- aflatoxigenic, cytotoxic	Food preservative	Food	[118]
*Myrtus communis Mentha pulegium*	Common myrtle, Peppermint	Shoots	-	Nanoemulsion	-	Nanoemulsions	Antimicrobial	Food preservative	Food	[119]
*Cinnamomum* spp.	Cinnamon	-	-	Nanoemulsion	-	Nano- emulsions	-	-	Food	[120]
*Satureja kermanica*	Savory	Leaves	thymol (46.54%); carvacrol (30.54%); γ-terpinene (6.58%)	Nano- precipitation	Chitosan- cinnamic acid	Nanogels	Antifungal	-	Food	[121]
*Cymbopogon* *martinii*	Palmarosa	Leaves	geraniol (19.06%); geraniol (14.84%); geranyl propionate (12.88%)	Nano- precipitation	Chitosan	Nanocapsules	Antifungal	Food preservative	Food	[122]
*Syzygium* sp.	Cloves	-	-	Nanoemulsion	Gelatin, pullulan, inulin	Nanoemulsions	Antibacterial	Food preservative	Food	[123]
*Thymus vulgaris*	Thyme	-	thymol (43.63%); *p*-cymene (22.86%); bornyl acetate (8.70%)	Nanoemulsion	-	Nanoemulsions	Antimicrobial	Food preservative	Food	[124]
*Origanum vulgare Thymus capitatus*	Oregano Thyme	Aerial parts	thymol (43%); γ-terpinene (15%) and *p*-cymene (14%)	Nano- precipitation	Poly (ε-caprolactone)	Nanocapsules	Antibacterial	-	Pharmaceutical, food	[71]
*Origanum* *glandulosum*	Oregano	Aerial parts	carvacrol (26.29%); γ-terpinene (23.43%); thymol (19.52%)	High-speed homogenization, high-pressure homogenization	Sodium alginate	Nanocapsules Nanoemulsions	Antioxidant, anticancer	-	Pharmaceutical, food	[80]
*Cinnamomum zeylanicum* *Thymus vulgaris Schinus molle*	Cinnamon, Thyme, Peruvian peppertree	Leaves	-	Ionic gelidification	Chitosan	Nanocapsules	Antimicrobial	-	Pharmaceutical, food	[85]
*Aniba canelilla*	Preciosa	Leaves and branches	1-nitro-2-phenylethane (86.63%); methyleugenol (12.7%); benzaldehyde (0.663%)	Nanoemulsion	-	Nanoemulsions	Anti- Chemotactic	Healing of infected wounds	Pharmaceutical	[46]
*Eugenia* *caryophyllata*	Cloves	Flower buttons	eugenol (89.86%); β-caryophyllene (5.40%)	Ionic gelidification– emulsion	Chitosan	Nanoparticles	Antioxidant, antibacterial	Preservative, Medicine	Pharmaceutical, cosmetic	[60]
*Thymus vulgaris*	Thyme	-	thymol (22.10%); *p*-cymene (21.31%); carvacrol (13.02%)	High-pressure homogenization		Nanoemulsions	Antifungal	Healing of infected wounds	Pharmaceutical	[75]
*Homalomena* *pineodora*	-	Leaves	-	Ionic gelidification	Chitosan	Nanocapsules	Antimicrobial	Healing of diabetic ulcers	Pharmaceutical	[76]
*Morinda citrifolia*	Indian mulberry	Seeds	nordamnacanthal (22.34%); α-copaene (22.96%); α-morenone (20.45%)	Nano- precipitation	Chitosan	Nanocapsules	Anticancer	-	Pharmaceutical	[78]
*Citrus aurantium Citrus limon Citrus sinensis*	Seville orange, Lemon, Sweet orange	-	Seville orange: sabinene (15.6%); ɣ-terpinene (6.0%); linalool (5.6%). Sweet orange: α-pinene (3.5%); sabinene (17%); trans-limonene oxide (3.1%). Lemon: trans-*p*-2,8- menthadien-1-ol (5.0%); cis-limonene oxide (2.6%); trans-limonene oxide (2.3%)	Ionic gelidification	Chitosan	Nanocapsules	Anticancer	-	Pharmaceutical	[79]
*Origanum vulgare Thymus capitatos*	Oregano Thyme	Aerial part	-	Ionic gelidification	Chitosan	Nanocapsules	Antimicrobial	Medicine	Pharmaceutical	[81]
*Cinnamomum* spp.	Cinnamon	Bark	-	Liposomes, lipid nanoparticles	Sodium alginate	Hybrid composite nanoparticles	Antimicrobial	Medicine	Pharmaceutical	[82]
*Citrus aurantifolia, Citrus hystrix, Citrofortunella microcarpa*	Lime, Makrut lime Calamondin	-	-	Spontaneous emulsification	-	Nanoemulsions	Antibacterial	Medicine	Pharmaceutical	[83]
*Poiretia latifolia*	Erva de touro	Leaves	trans-dihydrocarvone (15.3–51.2%); carvone (12.3–39.0%); limonene (13.9–29.4%)	Phase inversion	Soy lecithin	Lipossomes, Nanoemulsions	Antifungal, anti- Inflammatory, antioxidant	-	Pharmaceutical	[84]
*Eugenia* *caryophyllata*	Cloves	Aerial parts	-	High shear homogenization and ultrasound	-	Nanoemulsions	Antimicrobial	-	Pharmaceutical	[86]
*Cymbopogon* *commutatus*	Lemongrass	Whole plant	geranial (38.6%); neral (30.3%); geranyl acetate (8.2%)	Ionic gelidification- emulsion	Chitosan	Nanocapsules	Antimicrobial	-	Pharmaceutical	[87]
*Mentha pulegium*	Pennyroyal	-	pulegone (72.18%); piperitenone (24.04%); chrysanthenol (0.90%)	Hot melt homogenization	Nanostructured lipid carriers (NLC)	Nanogels	Antimicrobial	Healing of infected wounds	Pharmaceutical, cosmetic	[50]
*Mentha* × *piperita*	Peppermint	Leaves	menthol (39.80%); menthone (19.55%); neomenthol (8.82%)	Nanoemulsion	Nanostructured lipid carriers (NLC) and xanthan gum	Nanogels	Antimicrobial	Healing of infected wounds	Pharmaceutical, cosmetic	[74]
*Cynometra* *cauliflora*	Nam Nam	Leaves, branches, fruits	-	Ionic Gelidification– emulsion	Chitosan	Nanocapsules	Antimicrobial, antioxidant, cytotoxic		Pharmaceutical, cosmetic	[77]
*Cedrus deodara*	Cedar	Sawdust	α-cedarene (32.72%); β-cedarene (12.26%); thujopsene (24.03%)	Nanoemulsion	Modified starch	Nanoemulsions	Antioxidant, antibacterial	Preservative, Medicine	Pharmaceutical, cosmetic	[89]
*Thymus vulgaris Melissa officinalis*	Thyme, Lemon balm, Black caraway	-	-	Phase inversion	Sunflower oil	Nanoemulsions	Antioxidant, antibacterial	-	Pharmaceutical, cosmetic	[90]
*Mentha* × *piperita Camellia sinensis*	Peppermint, Green tea	-	-	Nano- precipitation	Chitosan	Nanocapsules	Antimicrobial, antioxidant	-	Pharmaceutical, cosmetic	[91]
*Syzygium* *aromaticum*	Cloves	Flower buds	eugenol (71.92%); β-caryophyllene (22.80%); chavibetol acetate (2.89%)	Intercalation	Bentonite clay and polyvinylpyrrolidone (PVP)	Nano- composites	Cytotoxic, Larvicide	-	Pharmaceutical, environmental	[51]
*Ocimum basilicum*	Basil	Leaves	trans-β-guaiene (16.89%); α-cadinol (15.66%); phytol (11.68%)	Nanoemulsion	-	Nanoemulsions	Antioxidant, antibacterial, larvicide	-	Pharmaceutical, environmental	[105]
*Satureja hortensis*	Summer savory	Aerial parts	carvacrol (35.2%); γ-terpinene (17.6%); thymol (12.1%)	Ionic gelidification	Chitosan	Nanocapsules	Acaricide	-	Environmental	[107]
*Cymbopogon* *nardus*	Citronella grass	-	-	Ionic gelidification	Chitosan and cellulose	Nanocapsules	Insecticide	Pest control	Environmental	[103]
*Mentha* × *piperita*	Peppermint	Dried leaves	l-menthone (32.27%); menthol (23.47%); α-phellandrene (7.71%)	Ionic Gelidification– emulsion	Chitosan	Nanocapsules	Insecticide	Pest control	Environmental	[104]
*Siparuna* *guianensis*	Negramina	Whole plant	-	Nanoemulsion	Chitosan	Nanocapsules	Larvicide	Pest control	Environmental	[106]
*Cannabis sativa*	Marijuana	Aerial parts	(E)-caryophyllene (23.1%); α-pinene (15.8%); myrcene (14.5%)	Nano- precipitation	Alfalfa protein	Nanocapsules	Antioxidant	-	Cosmetic, food	[92]
*Cymbopogon* *densiflorus*	Lemongrass	Leaves	trans-*p*-mentha-2,8-dien-1-ol (13.13%); cis-*p*-mentha-2,8- dien-1-ol (17.29%); trans-*p*-mentha-1(7),8-dien-2-ol (18.99%)	Phase inversion	-	Nanoemulsions	Antioxidant	-	Cosmetic	[125]

^a^ Part of the plant used in the extraction of the essential oil.

## 5. Encapsulated EOs versus Non-Encapsulated EOs

The advantages of using encapsulated EOs have recently been demonstrated in studies comparing the use of encapsulated and non-encapsulated oils. Through this evidence, the benefits of using encapsulation methods are clear, since they protect these bioproducts from environmental influences (decomposition by heat, humidity, light and oxygen), reduce volatility, improve stability, thus promoting a longer life, in addition to providing controlled release, which prolongs the biological effect of the compounds [46].

Mukurumbira et al. [126] conducted an extensive review of the effects of in natura and encapsulated essential oils on food contamination by harmful and pathogenic microorganisms. According to the authors, although essential oils are potent antimicrobials, they are chemically and biologically unstable and have strong aromas that limit their application as additives in food. Various encapsulation methods are increasingly being explored as a way to stabilize essential oils, mask their aromas, and possibly enhance their antimicrobial activity with a more sustained release of antimicrobials. The authors also mention the evidence of the greater effectiveness of encapsulated essential oils compared to non-encapsulated essential oils.

Barros et al. [127] evaluated the effect of the application of en-capsulated and non-encapsulated thyme essential oil on the mortality and persistence of the pest *Sitophilus zeamais* on the quality of corn grains during storage. The study showed that insect mortality was dependent on the concentration and time of exposure to the EO, and that the encapsulated essential oil was the most efficient in combating the insect. In addition, encapsulated EOs did not alter the quality characteristics of corn grains.

Singh et al. [128] conducted an extensive review of the action of essential oils as inhibitors of fungal infestation, their mode of action against fungal growth and the production of mycotoxins. The authors cited the use of nanoencapsulation as a promising new technology for protection of plant-based raw materials (herbal raw materials—HRMs). The use of formulations based on essential oils has been recommended as a green alternative to synthetic preservatives, since they are safer and more environmentally friendly. Nanoencapsulation maintains the stability of EOs and facilitates controlled delivery with improved maintenance of HRMs bioactive ingredients, which can boost the pharmaceutical, food and cosmetic sectors.

Milagres de Almeida et al. [129] studied the bacteriostatic and bactericidal effects of the essential oils of oregano, thyme, cloves, cinnamon and black pepper against strains of *Staphylococcus aureus*, *Listeria* sp., *Escherichia coli* and *Salmonella* sp., which are agents that contaminate food and cause foodborne illness. The study was conducted with encapsulated EOs and unencapsulated EOs and evaluated the synergistic effect between them. The encapsulation of the EOs of oregano, thyme and cloves was performed with different wall materials obtained by complex coacervation between three different polymers (chitosan, gelatin and gum arabic). The authors observed that encapsulation positively influenced the inhibitory power of the oils by resulting in minimal inhibitory concentrations (MICs), which were lower than those of the oil in an unencapsulated form. It was also proven that the encapsulation potentiated the effect of the EOs evaluated.

## 6. Conclusions

Currently, EOs are being investigated with prominent intensity, which brings about discoveries of relevant biological properties and the development of environmentally friendly bioproducts. In addition, this demand leverages the need for new technologies to preserve the stability, bioactivity and bioavailability of these substances, and the nanoencapsulation of EOs has been explored as an efficient approach to address such constraints.

In this review, an overview of the techniques, chemical composition and applications of polymer nanoparticles loaded with EOs, prepared via different encapsulation methods and different wall materials, as well as different bioactive elements, was emphasized.

In light of these data, the scenario points to a significant biotechnological potential of nanoencapsulated EOs. However, for future prospects of industrial applications more tests are needed, especially in vivo, in order to provide safe and unquestionable results. In addition, it is essential to develop studies that seek the production of nanoencapsulated EOs on a large scale, in order to meet the demands of the sectors in which they are applied.
